# Retraction: Correlation analysis between required surgical indexes and complications in patients with coronary heart disease

**DOI:** 10.3389/fsurg.2023.1237609

**Published:** 2023-06-19

**Authors:** Frontiers Editorial Office

A Retraction of the Original Research Article Correlation analysis between required surgical indexes and complications in patients with coronary heart disease by Tao M, Yao X, Sun S, Qin Y, Li D, Wu J, Xiong Y, Teng Z, Zeng Y and Luo Z (2022) Front. Surg. 9:948666. doi: 10.3389/fsurg.2022.948666

Following publication, the publisher uncovered evidence that several false identities were used in the peer-review process. The assignment of false reviewers was confirmed by an investigation conducted in accordance with Frontiers' policies and the Committee on Publication Ethics (COPE) guidelines.

The investigation also uncovered concerns about the presentation and validity of the data in the article. The authors did not respond to contact regarding the data presented in the article.

This retraction was approved by the Chief Editors of Frontiers in Surgery and the Chief Executive Editor of Frontiers.

The authors did not respond to contact regarding this retraction.

